# Supplementary material for: Lanthanide(III) Ions and 5-Methylisophthalate Ligand Based Coordination Polymers: An Insight into Their Photoluminescence Emission and Chemosensing for Nitroaromatic Molecules
Source: Nanomaterials (Basel). 2022 Nov 11;12(22):3977. doi: 10.3390/nano12223977 (PMC9694308; doi:10.3390/nano12223977)
Supplement: Supplementary file 1 [file nanomaterials-12-03977-s001.zip › nanomaterials-2002161-supplementary.pdf]

Electronic Supplementary Information for:

Lanthanide(III) Ions and 5-methylisophthalate Ligand Based Coordination Polymers: An Insight into their Photoluminescence Emission and Chemosensing for Nitroaromatic Molecules

Oier Pajuelo-Corral,‡[a] Laura Razquin-Bobillo,‡[a] Sara Rojas,[b] Jose Angel García,[c] Duane Choquesillo-Lazarte,[d] Alfonso Salinas-Castillo,[e] Ricardo Hernández,[a] Antonio Rodríguez-Diéguez[b,]* and Javier Cepeda[a,]*

[a] Departamento de Química Aplicada, Facultad de Química, Universidad del País Vasco (UPV/EHU), 20018 Donostia, Spain. [b] Dept. of Inorganic Chemistry, C/ Severo Ochoa s/n, University of Granada, 18071, Granada, Spain. [c] Departamento de Física, Facultad de Ciencia y Tecnología, Universidad del País Vasco/Euskal Herriko Unibertsitatea (UPV/EHU), 48940, Leioa, Spain. Donostia International Physics Center, Paseo Manuel de Lardizabal 4, 20018, Donostia, Spain. [d] Laboratorio de Estudios Cristalográficos, IACT, CSIC-Universidad de Granada, Avda. de las Palmeras 4, 18100 Armilla, Spain. [e] Departamento de Química Analítica, C/ Severo Ochoa s/n, University of Granada, 18071, Granada, Spain.

Contents:

S1. Structural details.

S2. Continuous Shape Measurements (CShMs).

S3. Thermogravimetric analysis.

S4. Powder X-ray Diffraction Analysis.

S5. FT-IR spectroscopy.

S6. Photoluminescence measurements and calculations.

S7. Sensing experiments.

S1. Additional structural details.

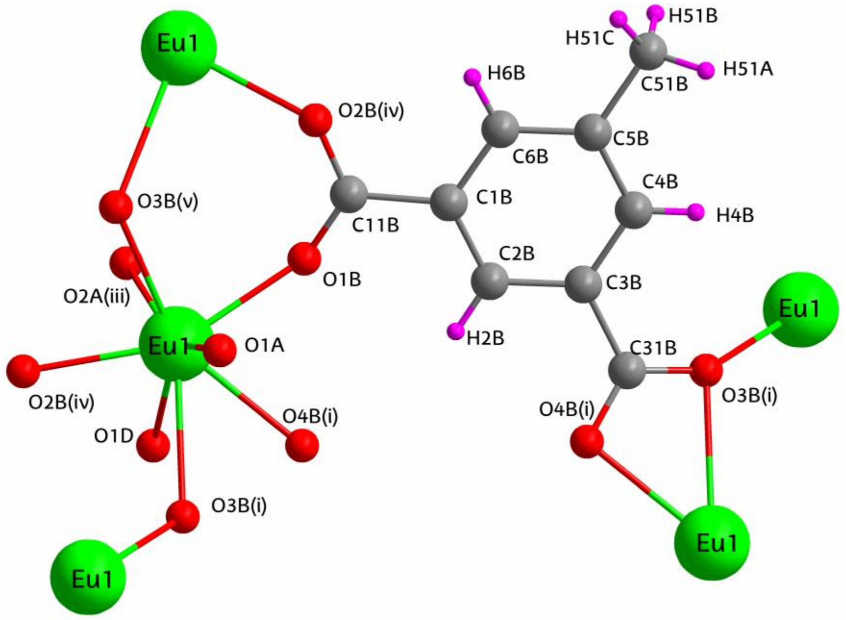

Figure S1. Representation of the bonds of ligand B, which are the same as in ligand A.

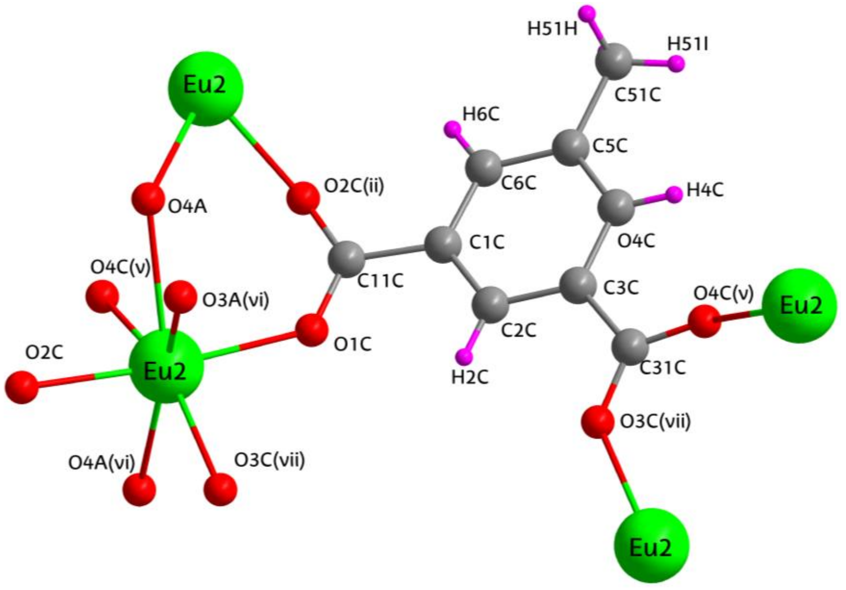

Figure S2. Representation of the C ligand bonds.

Crystallographic data have been deposited with the Cambridge Crystallographic Data Center as supplementary publication with CCDC numbers 2212971-2212972. Copies of the data can be obtained free of charge on application to the Director, CCDC, 12 Union Road, Cambridge, CB2 1EZ, U.K. (Fax: +44-1223-335033; e-mail: deposit@ccdc.cam.ac.uk or http://www.ccdc.cam.ac.uk).

S2. Continuous Shape Measurements (CShMs).

Table S1. Continuous Shape Measurements for the coordination environment for compounds **1_{Sm}** and **2_{Eu}**. The lowest SHAPE values for each ion are shown in bold blue, indicating best fits.

Codes:

OP-8	1 D_{8h}	Octagon
HPY-8	2 C_{7v}	Heptagonal pyramid
HBPY-8	3 D_{6h}	Hexagonal bipyramid
CU-8	4 O_h	Cube
SAPR-8	5 D_{4d}	Square antiprism
TDD-8	6 D_{2d}	Triangular dodecahedron
JGBF-8	7 D_{2d}	Johnson gyrobifastigium J26
JETBPY-8	8 D_{3h}	Johnson elongated triangular bipyramid J14
JBTPR-8	9 C_{2v}	Biaugmented trigonal prism J50
BTPR-8	10 C_{2v}	Biaugmented trigonal prism
JSD-8	11 D_{2d}	Snub diphenoid J84
TT-8	12 Td	Triakis tetrahedron
ETBPY-8	13 D_{3h}	Elongated trigonal bipyramid

Structure [ML8]	OP-8	HPY-8	HBPY-8	CU-8	SAPR-8	TDD-8	JGBF-9
**1_{Sm} (Sm1)**	26.530	21.690	14.320	11.111	2.115	2.805	13.047
**2_{Eu} (Eu1)**	26.517	21.854	14.345	11.076	2.047	2.781	12.978

Structure [ML8]	JETBPY-8	JBTPR-8	BTPR-8	JSD-8	TT-8	ETBPY-8
**1_{Sm} (Sm1)**	27.741	2.096	**1.986**	3.894	11.598	21.442
**2_{Eu} (Eu1)**	27.529	2.023	**1.936**	3.782	11.574	21.531

Codes:

HP-7	1 D_{7h}	Heptagon
HPY-7	2 C_{6v}	Hexagonal pyramid
PBPY-7	3 D_{5h}	Pentagonal bipyramid
COC-7	4 C_{3v}	Capped octahedron
CTPR-7	5 C_{2v}	Capped trigonal prism
JPBPY-7	6 D_{5h}	Johnson pentagonal bipyramid J13
JETPY-7	7C_{3v}	Johnson elongated triangular pyramid J7

**Structure [ML7]**	HP-7	HPY-7	PBPY-7	COC-7	CTPR-7	JPBPY-7	JETPY-7
**1_{Sm} (Sm2)**	30.630	24.240	**1.461**	7.256	5.716	4.408	20.905
**2_{Eu} (Eu2)**	30.794	24.265	**1.403**	7.177	5.615	4.290	20.892

S3. Thermogravimetric Analysis.

The thermal behaviour of compound **3Gd** has been studied as a representative compound for all isostructural counterparts by means of thermogravimetric/differential thermal analyses (TG/DTA) in order to further confirm the purity of the polycrystalline samples. As it can be seen in Figure S3, when the compounds are heated above room temperature, they show a small mass loss which is assigned to the loss of the coordinated DMF molecule (calc: 7.93 / exp: 7.00%, on the basis of the product lost). Above 400 °C, the compounds undergo an exothermic process as a consequence of the decomposition of organic ligand, rendering Gd_2O_3 as the final product (calc: 39.8 / exp: 38.9%, on the basis of the product remaining).

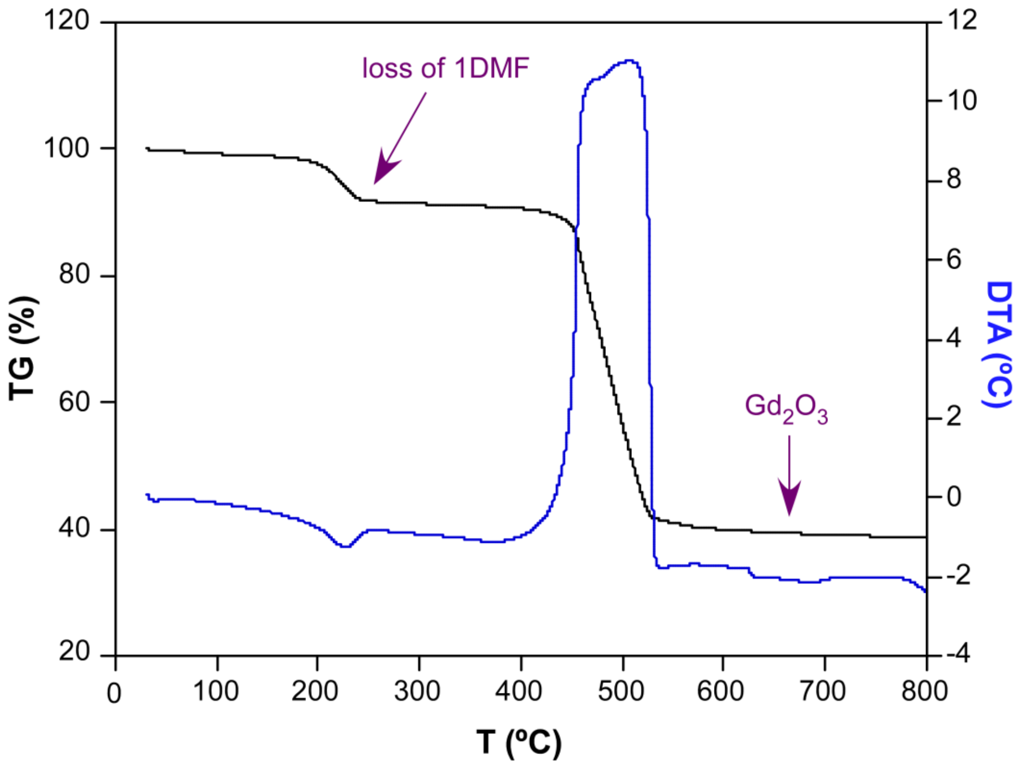

Figure S3. TG/DTA analysis for compound **3Gd**.

S4. Powder X-ray Diffraction Analysis.

Pattern-matching analysis confirms the purity of the polycrystalline samples of all compounds.

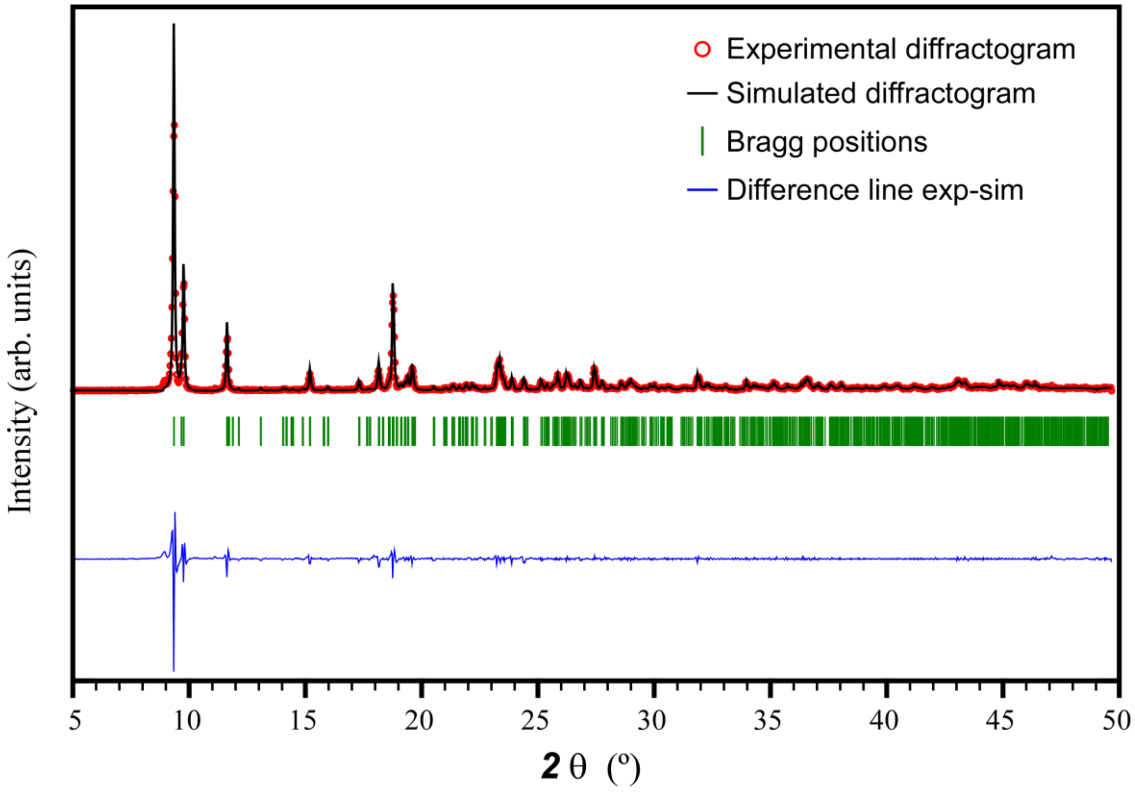

Figure S4. Pattern-matching analysis of polycrystalline sample of compound $\mathbf{1}_{\mathbf{Sm}}$.

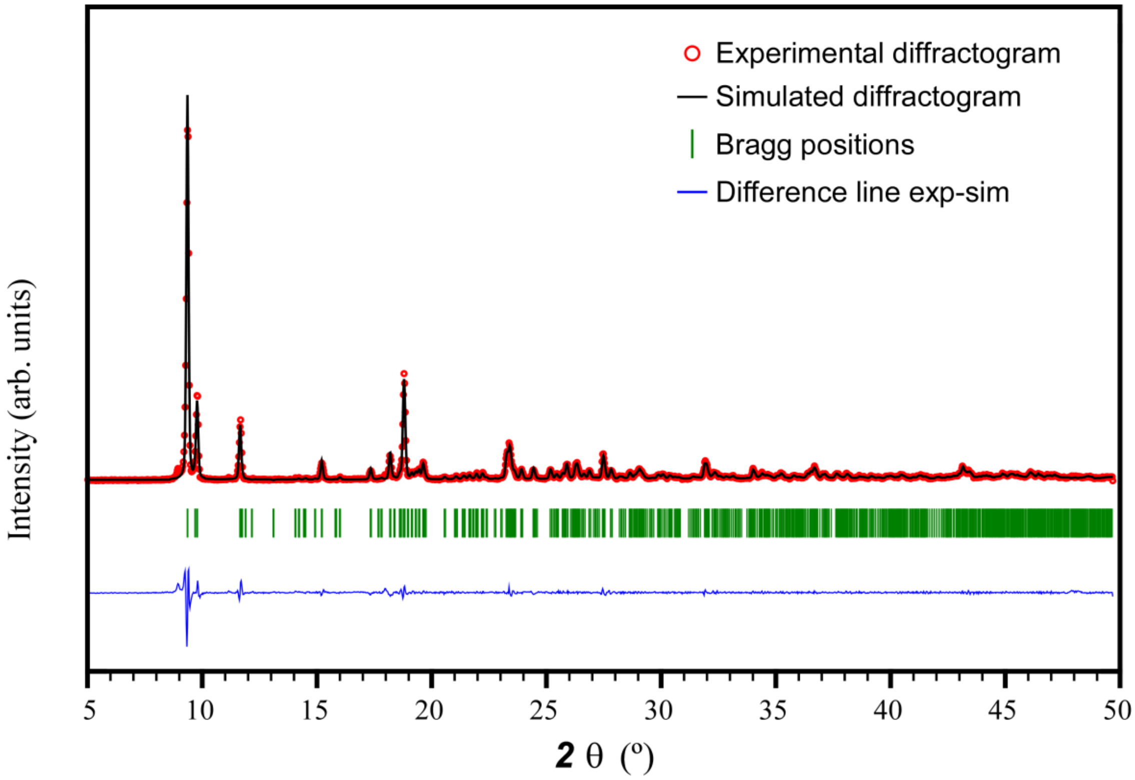

Figure S5. Pattern-matching analysis of polycrystalline sample of compound $\mathbf{2}_{\mathbf{Eu}}$.

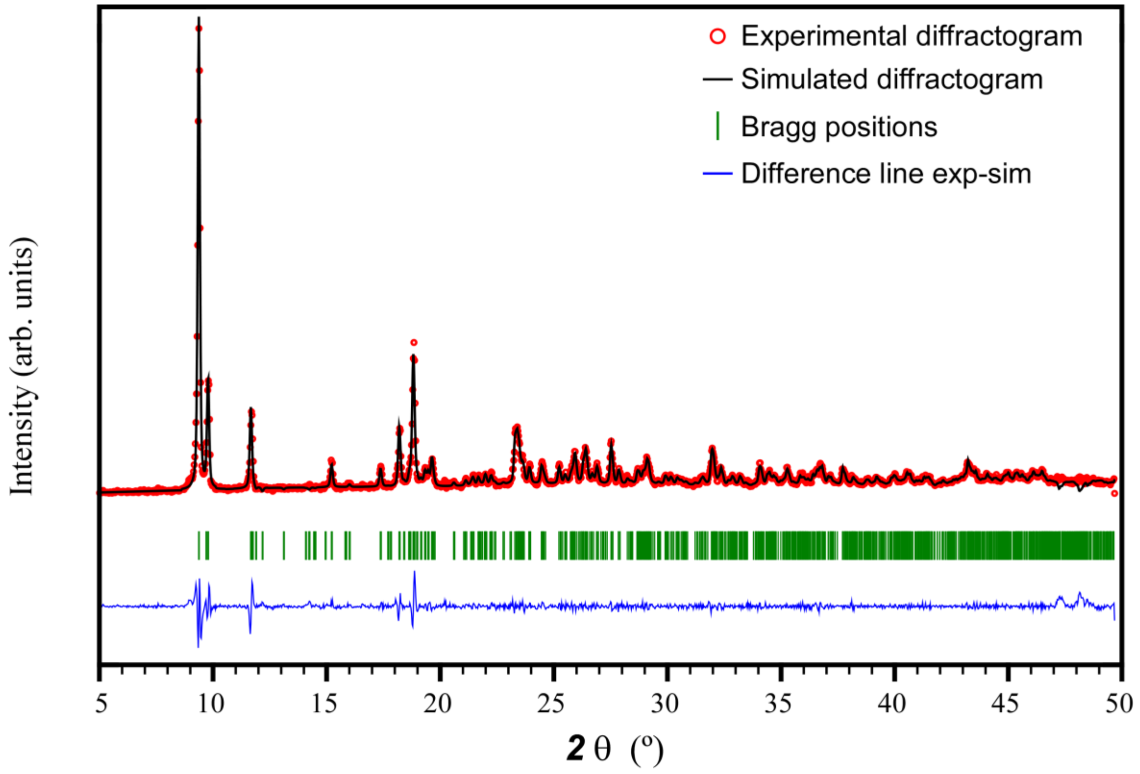

Figure S6. Pattern-matching analysis of polycrystalline sample of compound $\mathbf{3_{Gd}}$.

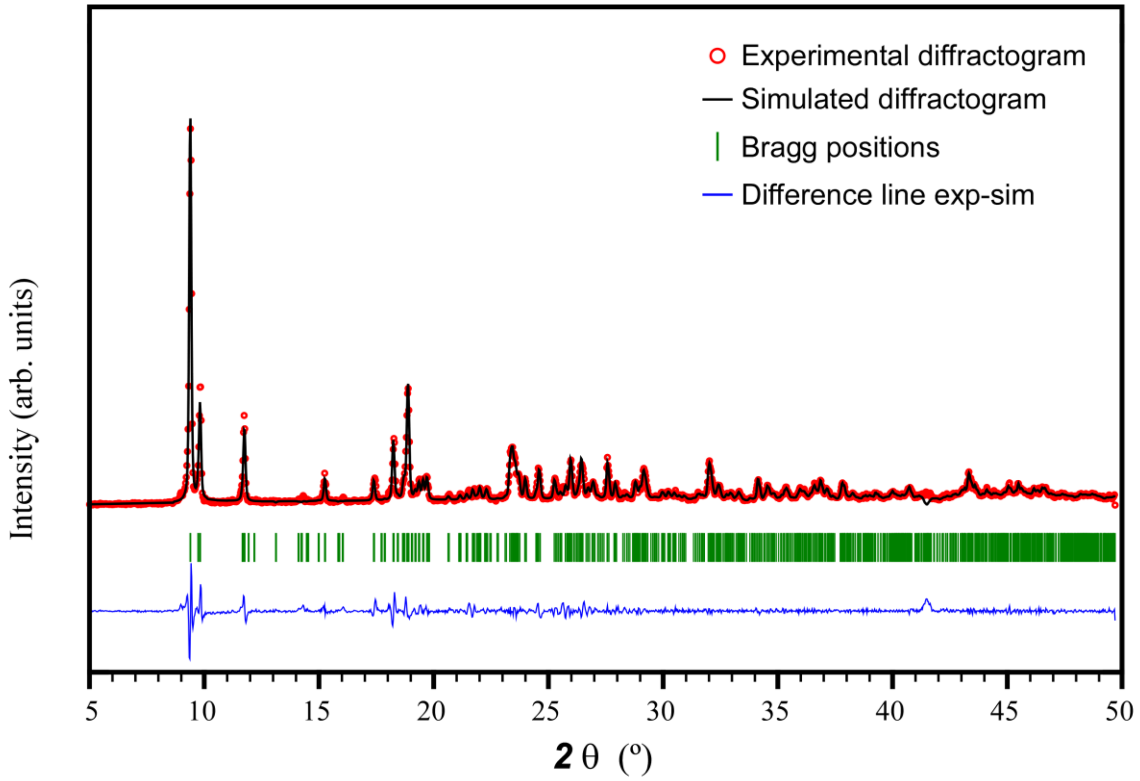

Figure S7. Pattern-matching analysis of polycrystalline sample of compound $\mathbf{4_{Tb}}$.

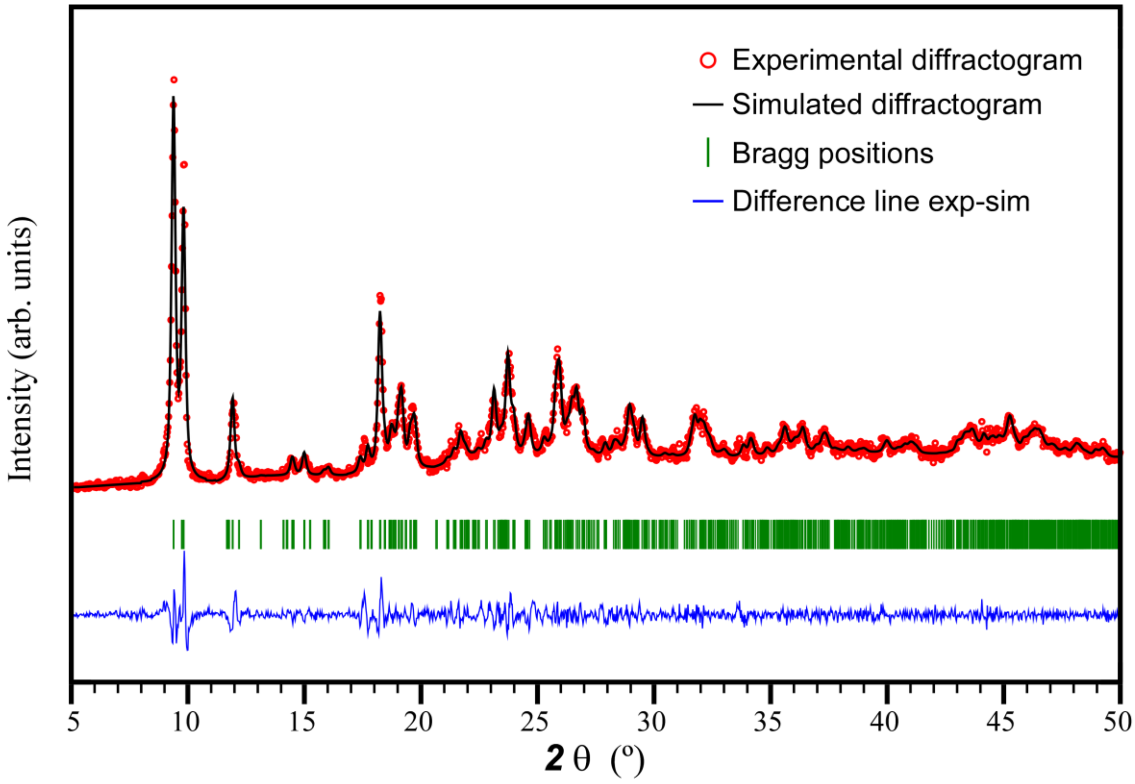

Figure S8. Pattern-matching analysis of polycrystalline sample of compound $\mathbf{5_{Yb}}$.

S5. FT-IR spectroscopy.

Infrared spectroscopy has been used for the initial characterization of the compounds, as it allows us to check whether the synthesized complexes contain the ligand and whether it is coordinated to the metals. The spectra obtained for compounds **1-5** show a similar structure (Figure S9).At high frequencies of FTIR spectrum, all compounds exhibit an intense band around 3600 cm^{-1} that corresponds to the vibration of O-H bond of the ligand. This band is followed by a second, broader band between 3300-2300 cm^{-1}, which corresponds to the vibrations of the O-H and C-H bonds of the ligand. The strong bands appearing in the range 1725-1450 cm^{-1} correspond to asymmetric vibrations of the carboxylate group, the C-O bond of the DMF molecule and the C-C bonds of the aromatic ring, while the symmetric vibrations of the carboxylate group appear in the range 1400-1200 cm^{-1}. The vibration bands of the M–O bonds (M = Sm, Eu, Gd, Tb, and Yb) are observed around the range of 605-400 cm^{-1}.

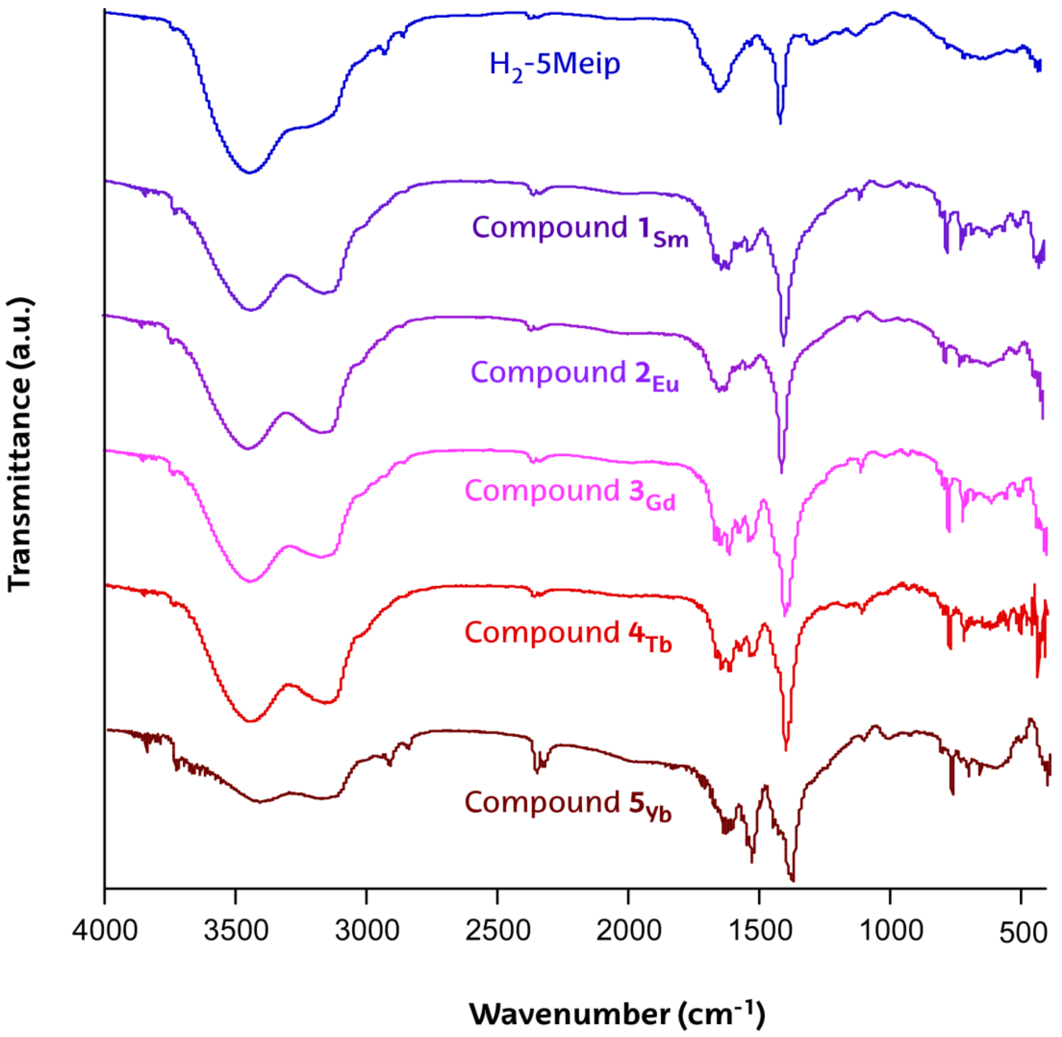

Figure S9. FTIR spectra of all compounds.

S6. Photoluminescence measurements and calculations.

First, the experimentally measured excitation and emission of the free ligand were compared to the spectra obtained from TDDFT calculations. As observed in the next figure, both spectra reproduce well the experimental ones. The main excitation (HOMO–1 → LUMO+4) and emission (HOMO ← LUMO+3) are the most representative transitions of the spectra, concluding that the photoluminescence proceeds through π-π* transitions.

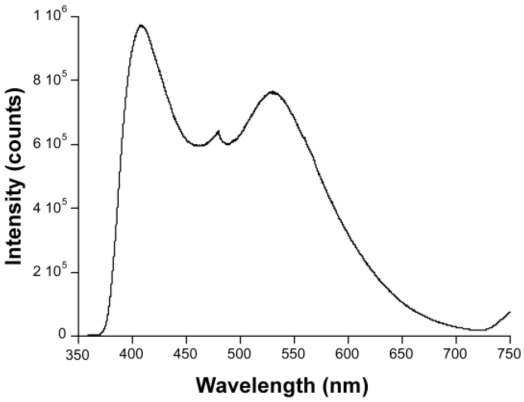

Figure S10. Emission spectra of the free H_25Meip ligand sample taken at room temperature at λ_{ex} = 325 nm.

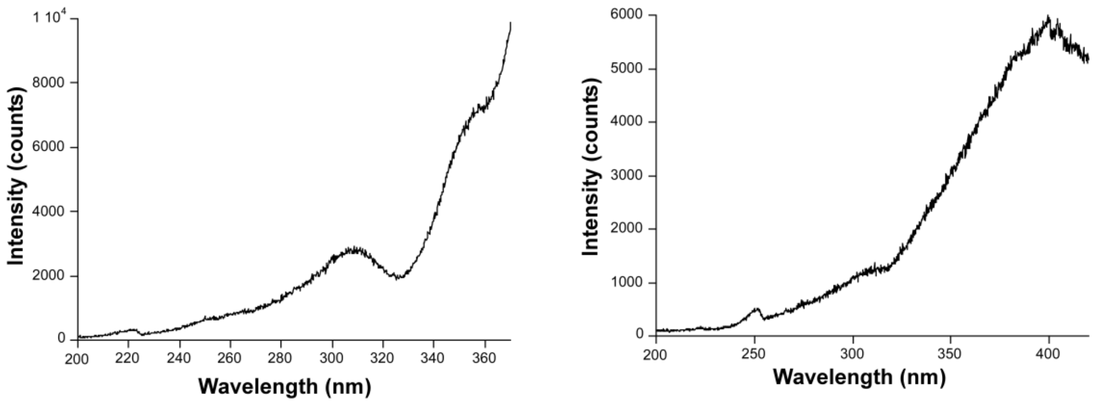

(a) **(b)**

Figure S11. Excitation spectra of the free H_25Meip ligand sample taken at room temperature: **(a)** λ_{em} = 408 and **(b)** 531 nm.

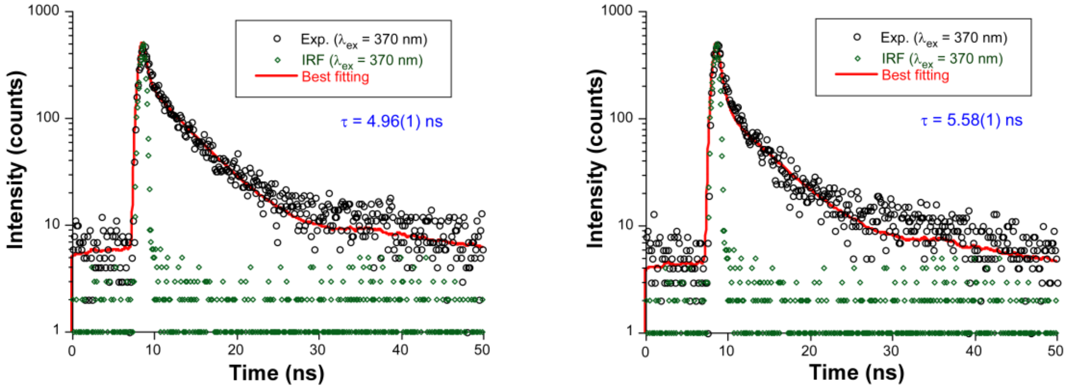

(a) **(b)**

Figure S12. Decay curves of the two emission maxima of free H_25Meip ligand sample taken at room temperature: **(a)** λ_{em} = 408 and **(b)** 531 nm.

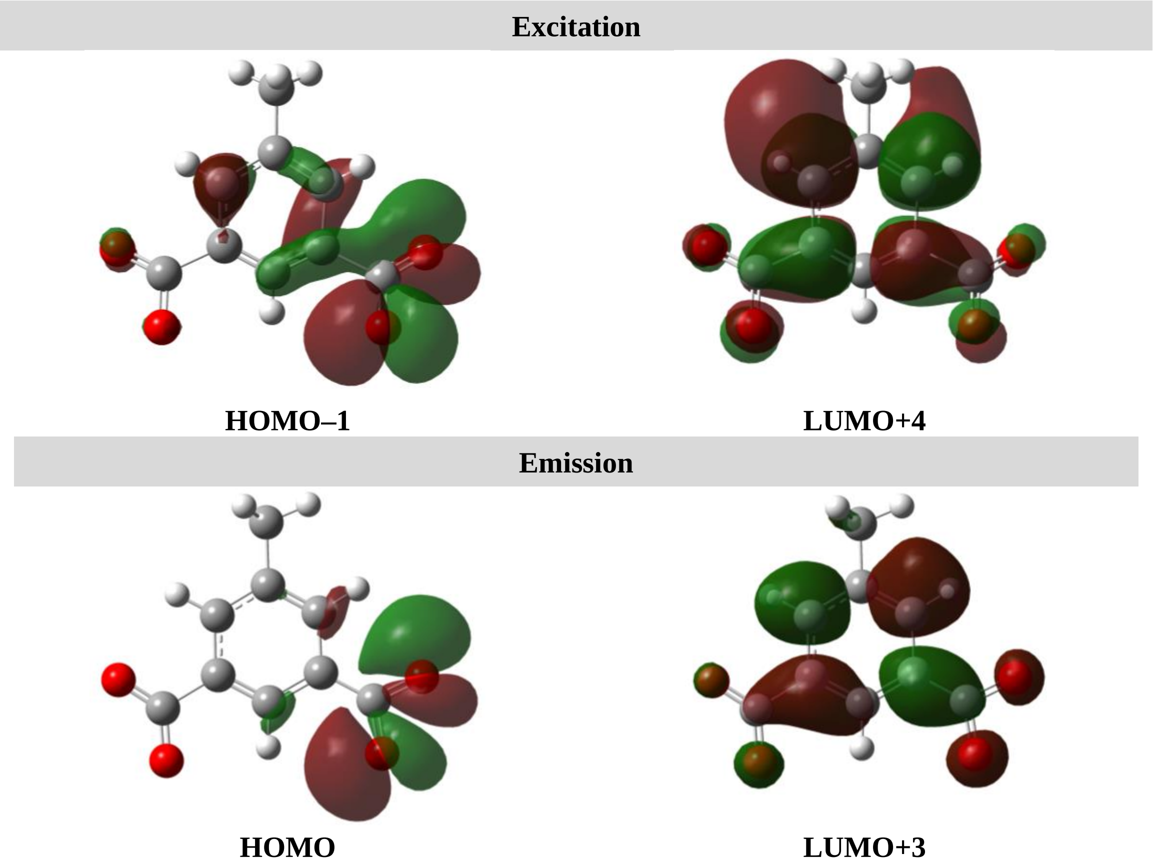

Figure S13. MOs of the H_25Meip ligand involved in the main excitation and emission.

In the case of the Gd-based model, the excitation takes place through a band centred 340 nm that is somewhat red shifted in the TDDFT computed spectrum. There, the main vertical line at 362 nm (HOMO–8 → LUMO+3) may be considered as the most representative transition for describing the process. It may be said that this transition possesses a similar π-π* nature described for the free ligand except for the fact that in $\mathbf{3_{Gd}}$ the HOMO–8 is quite extended over various ligands. Regarding the emission of the compound, the calculated emission spectrum does not reproduce well the experimental one, since the most intense bands (λ_{em} = 414 and 470 nm) seem to correspond to the less intense shoulders of the experimental spectrum (λ_{em} = 383 and 418 nm). The first vertical excitation corresponds to the HOMO–6 ← LUMO+3 whereas the second one is the HOMO–3 ← LUMO+2. It is worth noticing that the main experimental band centred at λ_{em} = 505 nm is not reproduced in this calculation.

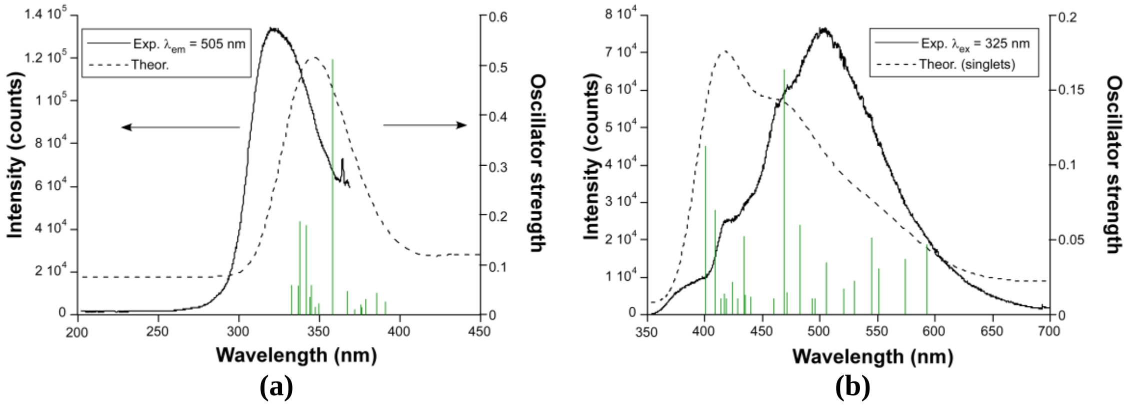

Figure S14. Experimental (solid line) and computed (dotted line) excitation and emission spectra of compound $\mathbf{3_{Gd}}$ taken at room temperature: **(a)** λ_{em} = 505 and **(b)** λ_{ex} = 325 nm. Vertical green bars represent the computed main vertical excitations.

Excitation

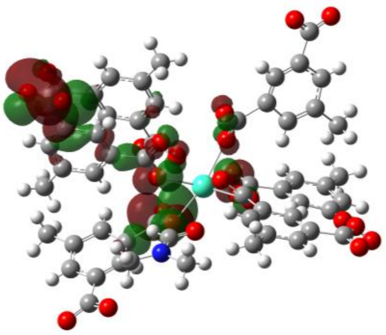

HOMO–8

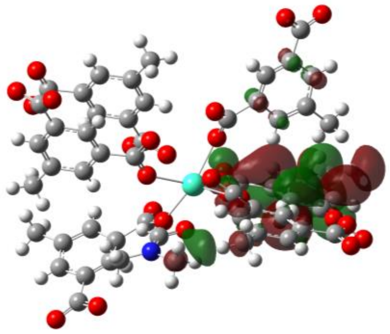

LUMO+3

Emission (λ_{em} = 414 nm)

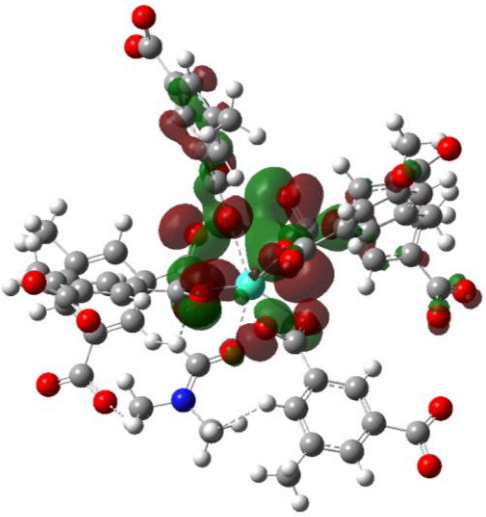

HOMO–6

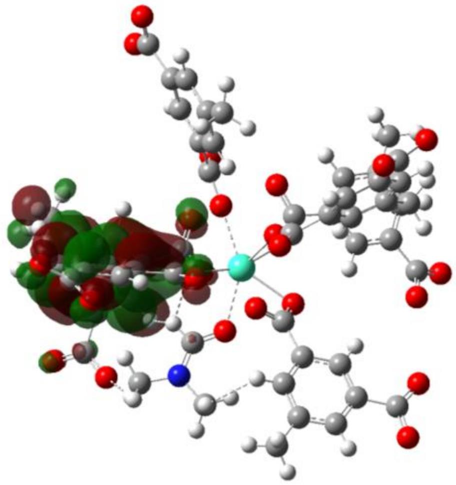

LUMO+3

Emission (λ_{em} = 470 nm)

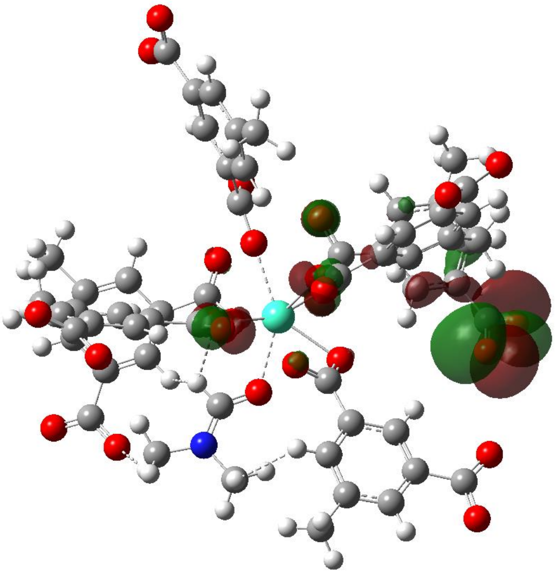

HOMO–3

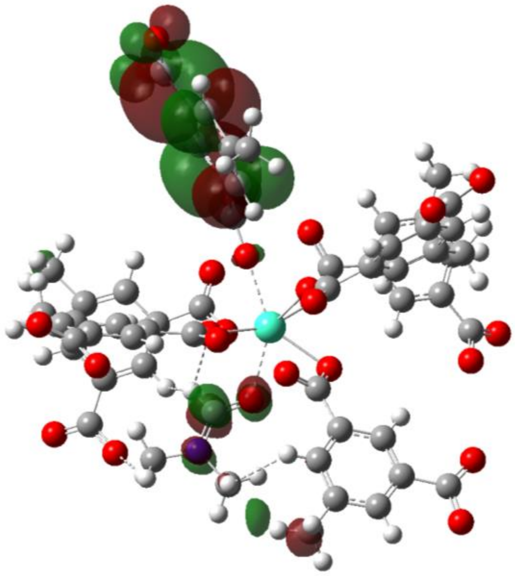

LUMO+2

Figure S15. MOs of compound **3_{Gd}** involved in the main excitation and emission calculated for singlet-to-singlet transitions.

To appropriately reproduce the emission spectra, we turned to the singlet-to-triplet methodology by which the emission is computed on the basis of the same absorption calculation. Using this approach, the calculated spectra presents a main band centred at 510 nm in addition to a shoulder 450 nm, which is very close to the main bands of the experimental spectrum. According to this calculation, the first emission band (described by the λ_{em} = 438 nm line) corresponds to the HOMO–2 ← LUMO+3, whereas the main emission (described by the λ_{em} = 502 nm line) is assigned to the HOMO–1 ← LUMO+2 transition. It is worth noticing that these molecular orbitals (in the triplet state) have similar shapes compared to those calculated for the ground singlet state.

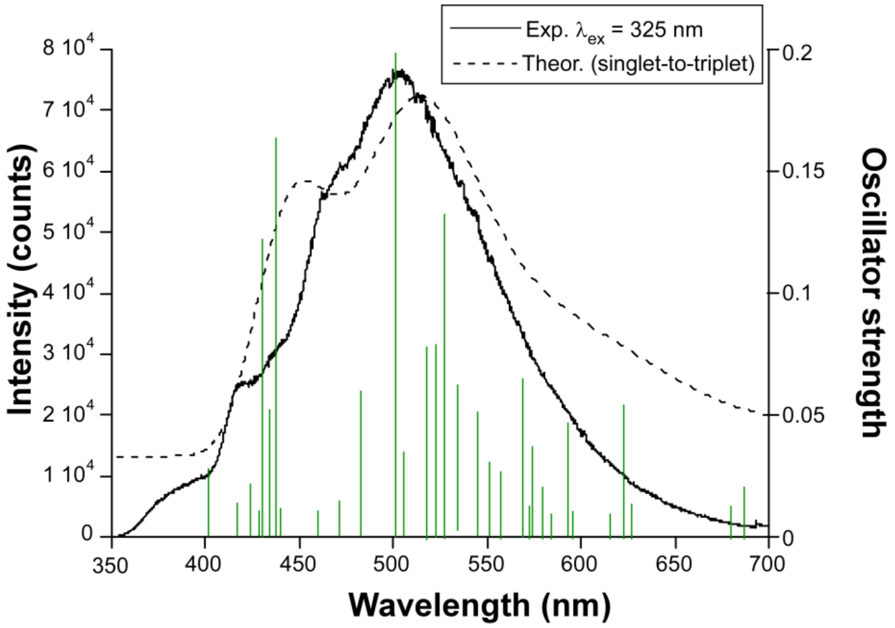

Figure S16. Experimental (solid line) and computed (dotted line) emission spectrum of compound **3_{Gd}** taken at room temperature. Vertical green bars represent the computed main vertical excitations.

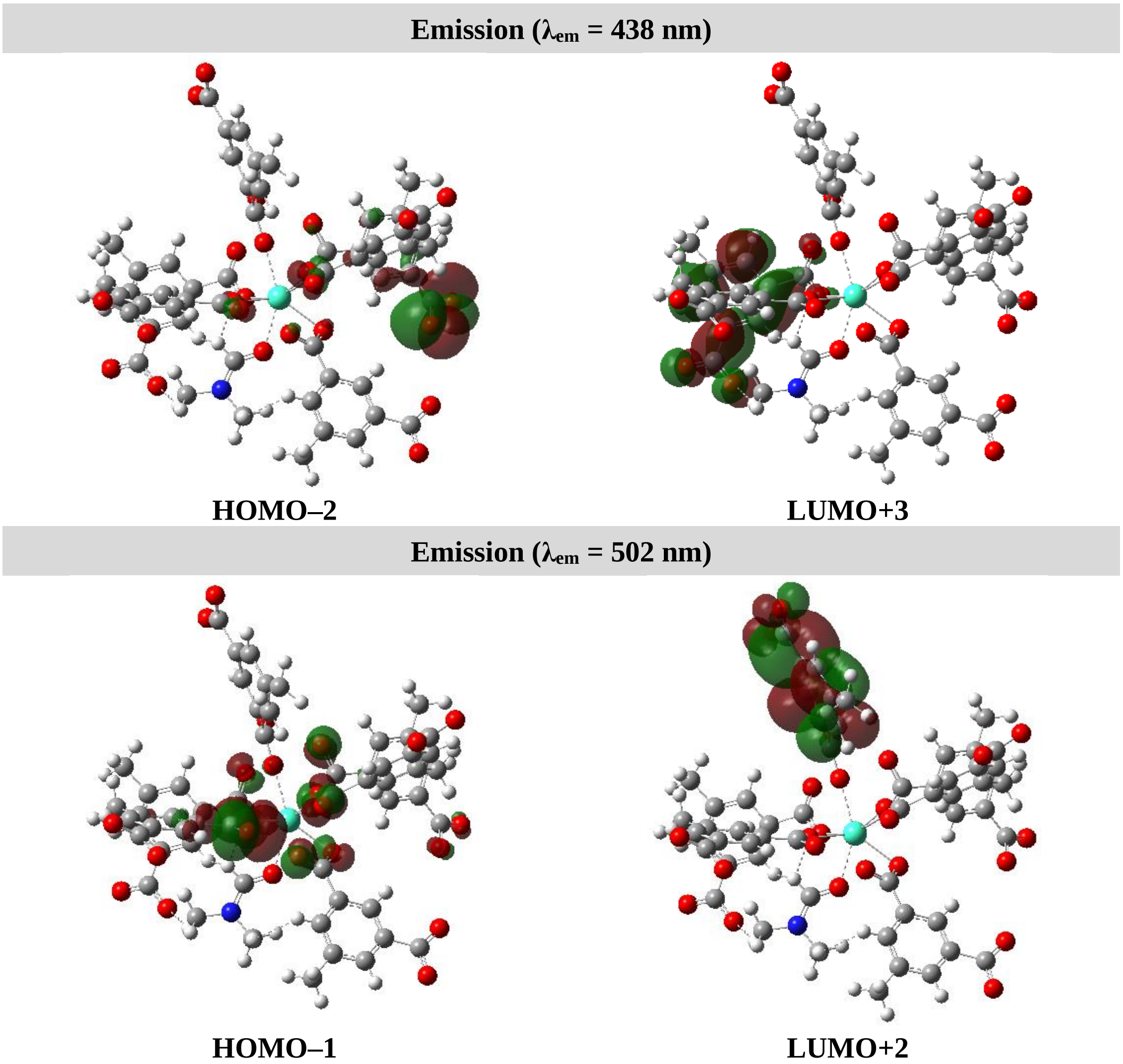

Figure S17. MOs of compound $\mathbf{3}_{\mathbf{Gd}}$ involved in the main excitation and emission calculated for singlet-to-triplet transitions.

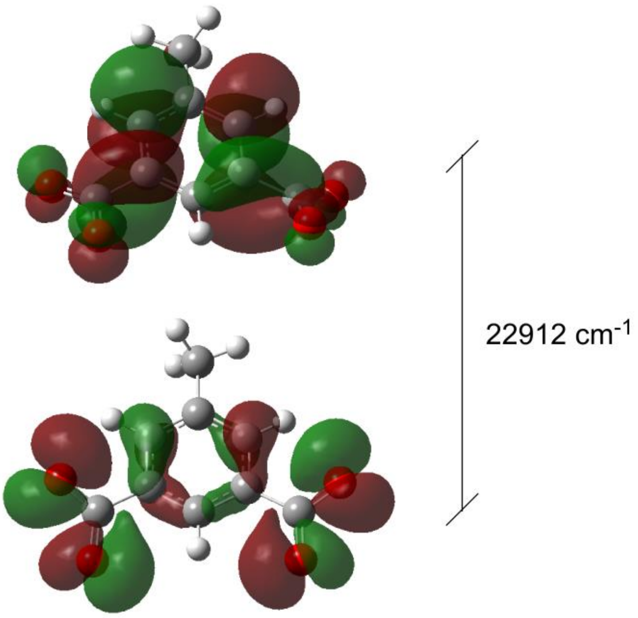

Figure S18. MOs of the ligand molecule involved in the triplet-to-singlet transition.

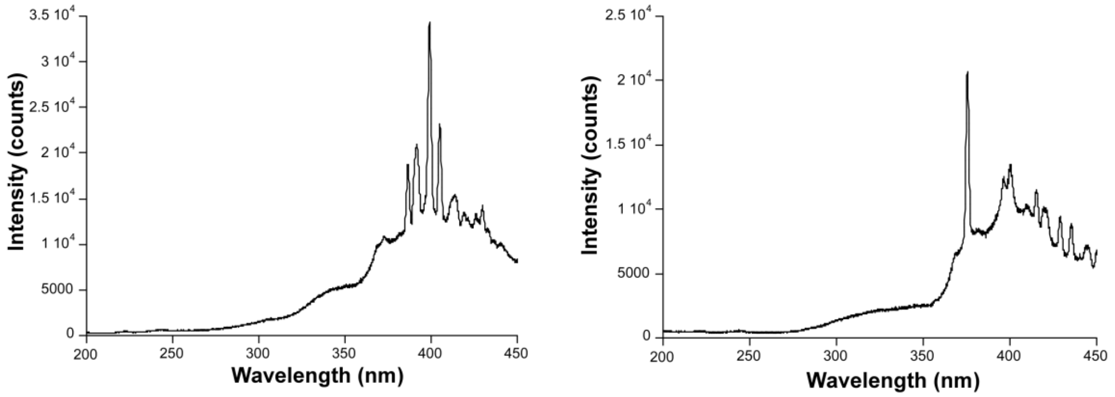

(a) **(b)**

Figure S19. Excitation spectra of compound $\mathbf{1_{Sm}}$ taken at room temperature under the main emission lines: **(a)** λ_{em} = 600 and **(b)** 646 nm.

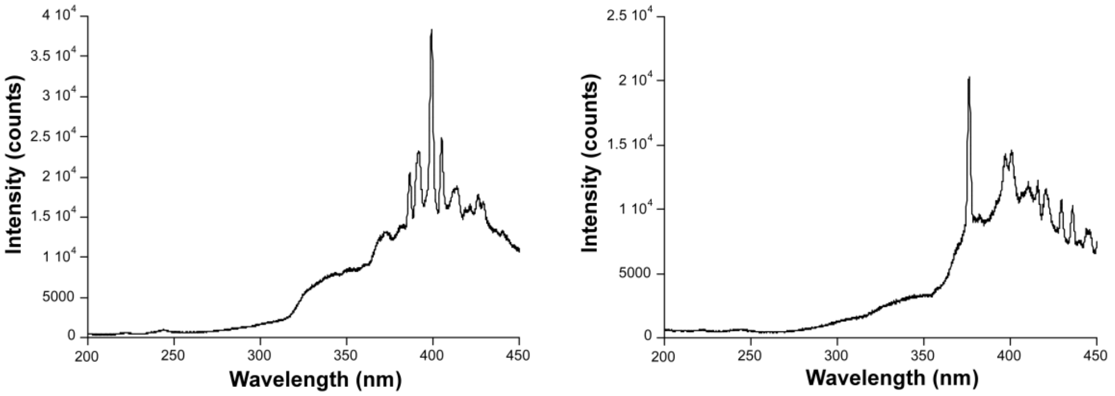

(a) **(b)**

Figure S20. Excitation spectra of compound $\mathbf{1_{Sm}}$ taken at 10 K under the main emission lines: **(a)** λ_{em} = 600 and **(b)** 646 nm.

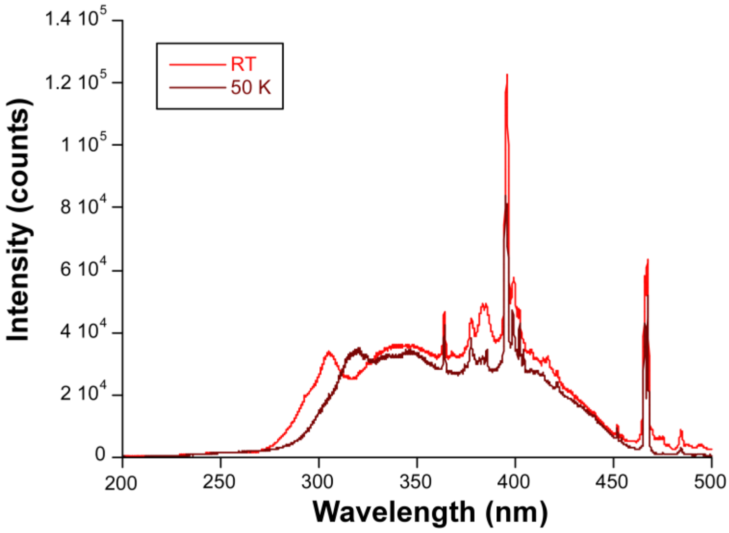

Figure S21. Excitation spectra of compound $\mathbf{2_{Eu}}$ recorded at the main emission line (λ_{em} = 613.8 nm) at variable temperature.

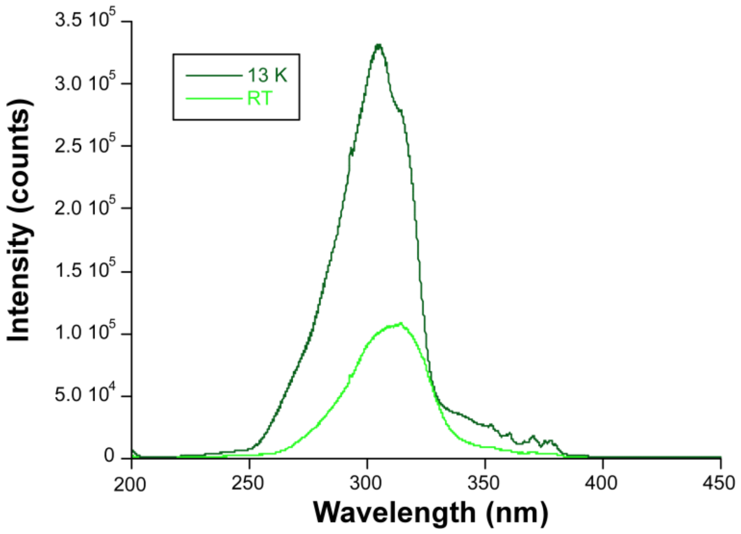

Figure S22. Excitation spectra of compound $\mathbf{4_{Tb}}$ recorded at the main emission line (λ_{em} = 542 nm) at variable temperature.

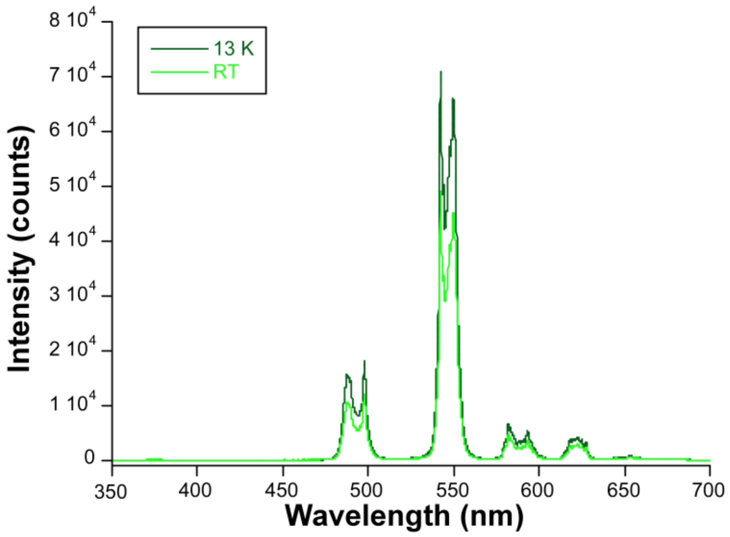

Figure S23. Comparison of the emission spectra of compound $\mathbf{4_{Tb}}$ recorded at the main emission line (λ_{ex} = 325 nm) at variable temperature.

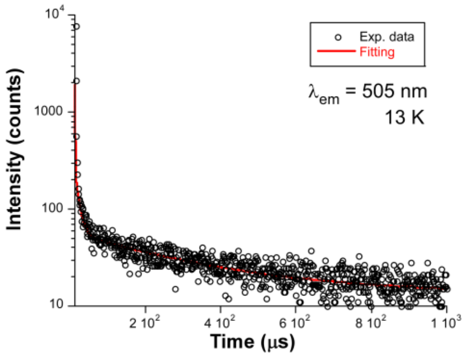

Figure S24. Decay curve of compound $\mathbf{3_{Gd}}$ taken at low temperature at the emission band maximum (λ_{em} = 505 nm).

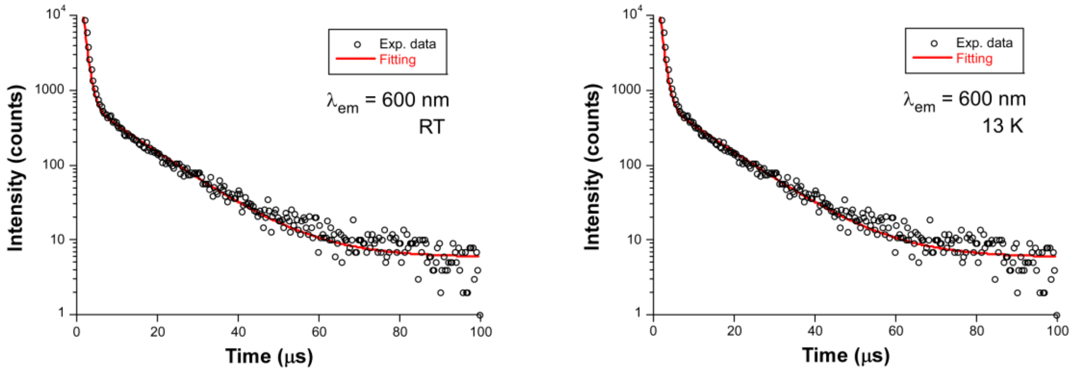

Figure S25. Decay curves of compound **1**$_{\mathbf{Sm}}$ taken at variable temperature under the main emission line (λ_{em} = 600 nm).

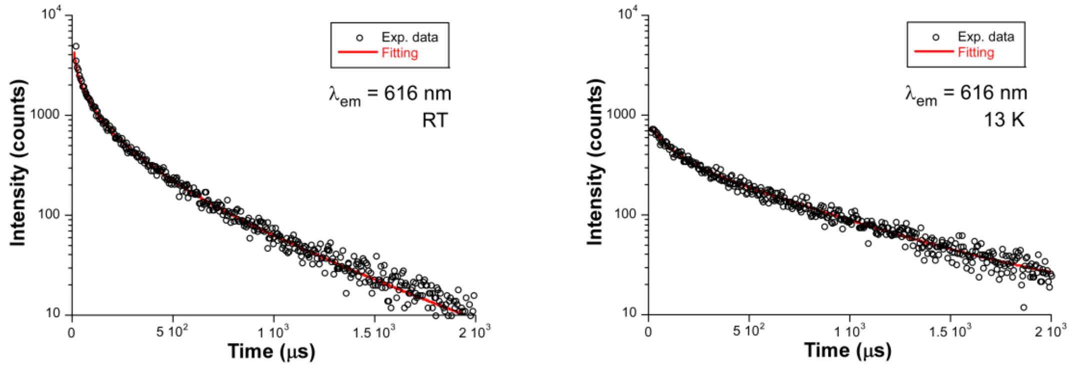

Figure S26. Decay curves of compound **2**$_{\mathbf{Eu}}$ taken at variable temperature under the main emission line (λ_{em} = 616 nm).

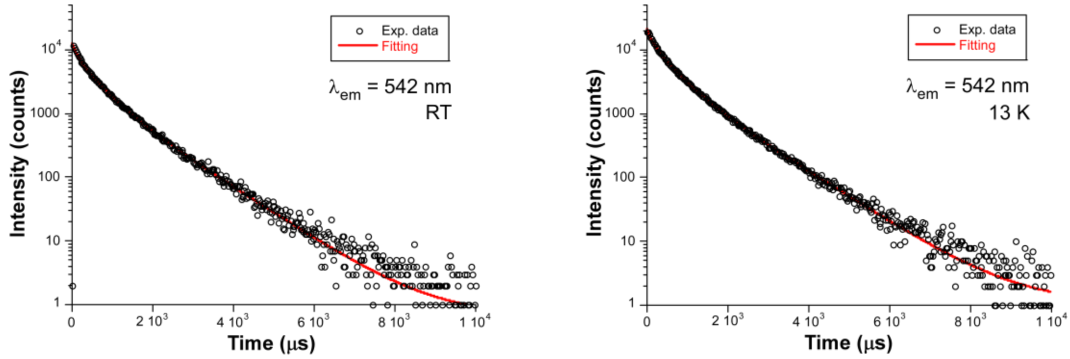

Figure S27. Decay curves of compound **4**$_{\mathbf{Tb}}$ taken at variable temperature under the main emission line (λ_{em} = 542 nm).

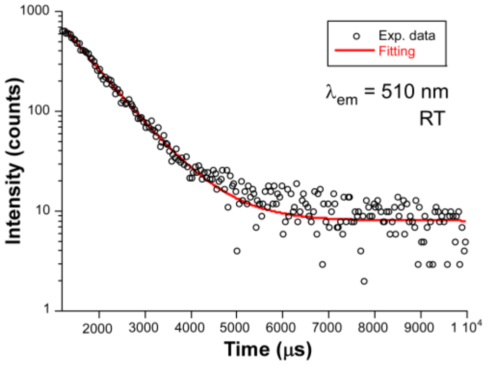

Figure S28. Decay curve of compound **1**$_{\mathbf{Sm}}$ taken at room temperature under the main line of the ligand emission (λ_{em} = 510 nm).

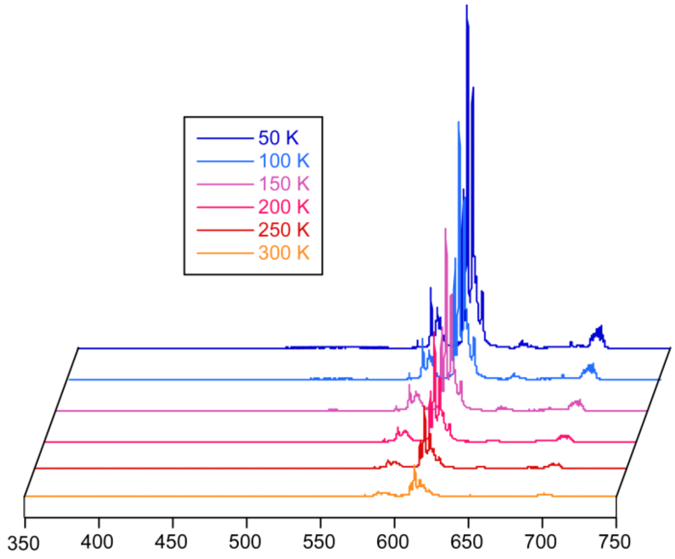

Figure S29. Variable temperature emission spectra of compound **2$_{Eu}$** under laser excitation (λ_{ex} = 325 nm).

Table S2. Spherical atomic coordinates, charge factors (*g*) and polarizabilities (*α*) for compounds **2D_Eu-L** and **3D_Eu-L**.

Eu1 centre/Atoms	Spherical coordinates			g	α (Å^3)
	R (Å)	Θ (°)	Φ (°)		
O1A	2.3001	125.79	57.35	1.2349	1.4723
O2A(i)	2.3535	78.19	345.57	1.2383	3.8744
O1B	2.4762	113.99	206.27	1.2328	0.8112
O2B(ii)	2.3351	37.16	213.62	1.2528	2.8518
O3B(iii)	2.4128	147.97	300.13	1.2384	0.0055
O3B(iv)	2.6564	100.51	274.58	1.2421	0.9944
O4B(iv)	2.4359	43.52	67.68	1.2403	2.8766
O1D	2.4034	88.38	140.39	1.2473	1.1410
**Eu2 centre/Atoms**	**Spherical coordinates**			**g**	**α (Å^3)**
	R (Å)	Θ (°)	Φ (°)		
O3A(v)	2.4256	91.16	230.79	0.5229	3.3804
O4A	2.3149	140.70	120.65	0.5251	3.0702
O4A(v)	2.6255	67.29	70.02	0.5230	0.6841
O1C	2.4293	93.20	25.09	0.5259	2.3952
O2C(vi)	2.2956	36.74	310.09	0.5236	2.4802
O3C(vii)	2.3421	131.03	302.24	0.5245	3.9452
O4C(viii)	2.3065	49.65	157.13	0.5240	2.6958

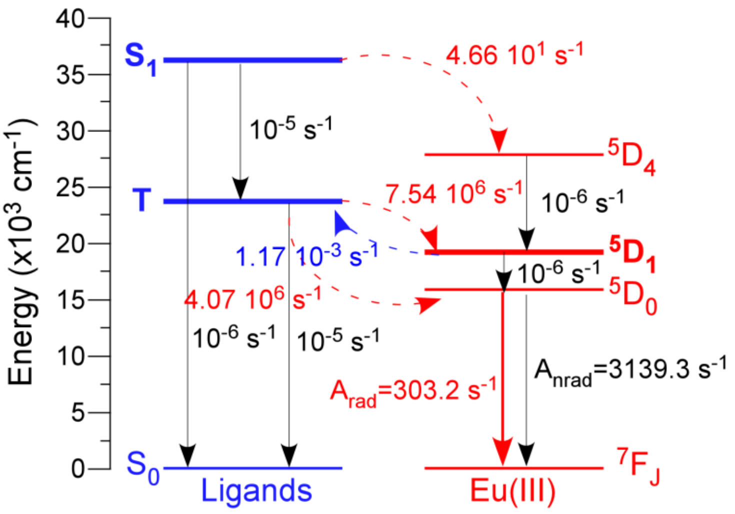

Figure S30. Schematic diagram of the main states involved in the luminescence of compound $\mathbf{2_{Eu}}$ using model 2-Eu-2 showing the main calculated energy transfer rates.

Note that in the case of the NIR-emitter (compound $\mathbf{5_{Yb}}$), the emission lifetime had to be estimated from deconvolution given the short and weak emission component involving the ligand fluorescence at room temperature. To that end, the measurement was performed with a diode laser which excites the sample at 370 nm.

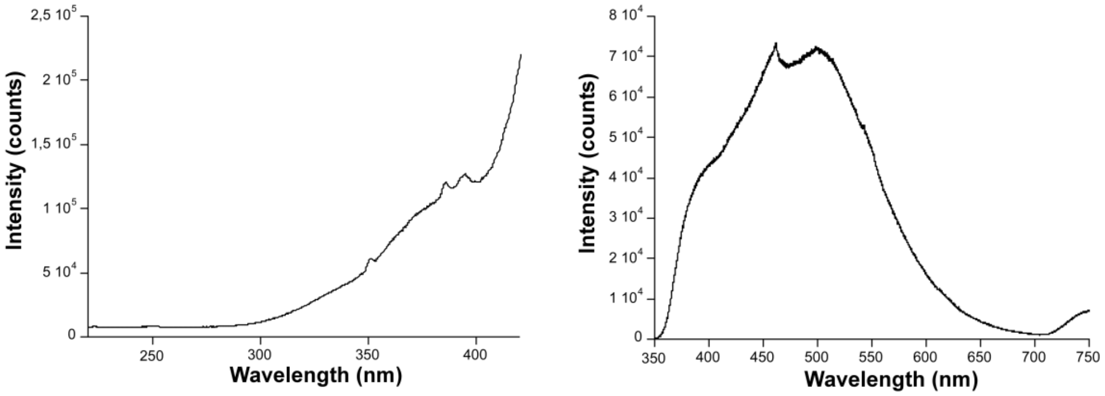

(a) **(b)**

Figure S31. Excitation and emission spectra of compound $\mathbf{5_{Yb}}$ taken at room temperature: **(a)** λ_{em} = 500 and **(b)** λ_{ex} = 325 nm.

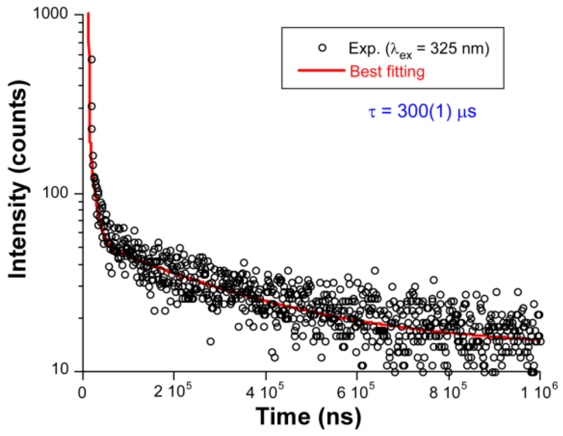

Figure S32. Decay curve of compound $\mathbf{5_{Yb}}$ measured at room temperature at the ligand emission (λ_{em} = 500 nm).

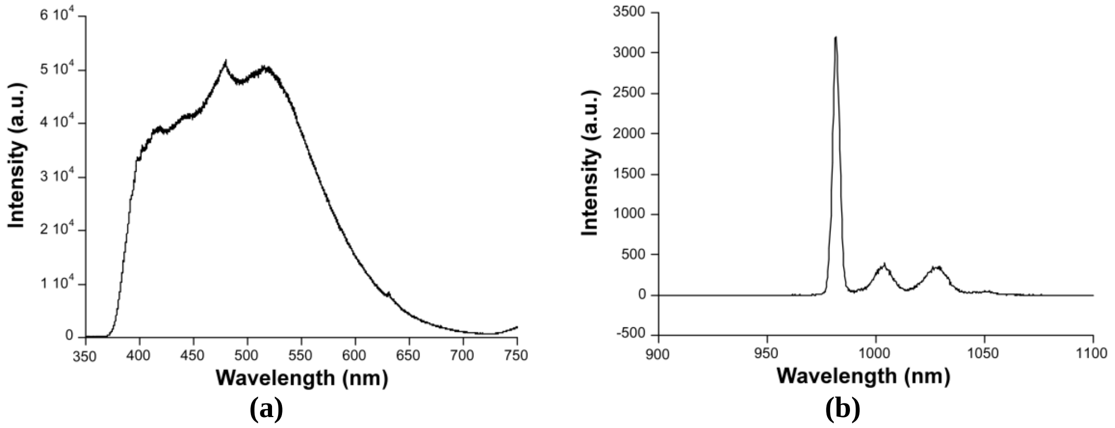

Figure S33. Emission spectra of compound $\mathbf{5_{Yb}}$ taken at 10 K showing the **(a)** visible and **(b)** NIR ranges.

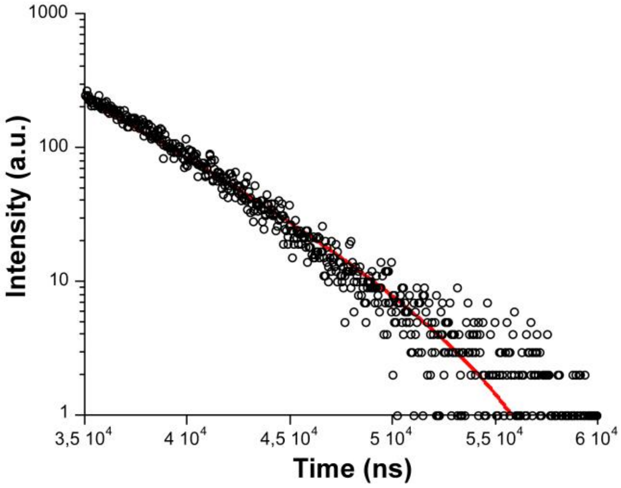

Figure S34. Decay curve of compound $\mathbf{5_{Yb}}$ measured at 10 K focusing the NIR emission line (λ_{em} = 980 nm).

Emission spectra of compounds $\mathbf{2_{Eu}}$ and $\mathbf{4_{Tb}}$ were also measured under normal conditions (under open atmosphere). Compared to those spectra recorded under high vacuum, they show an identical emission profile compared to that under high vacuum that only differs in a slight drop in the emission intensity for the former.

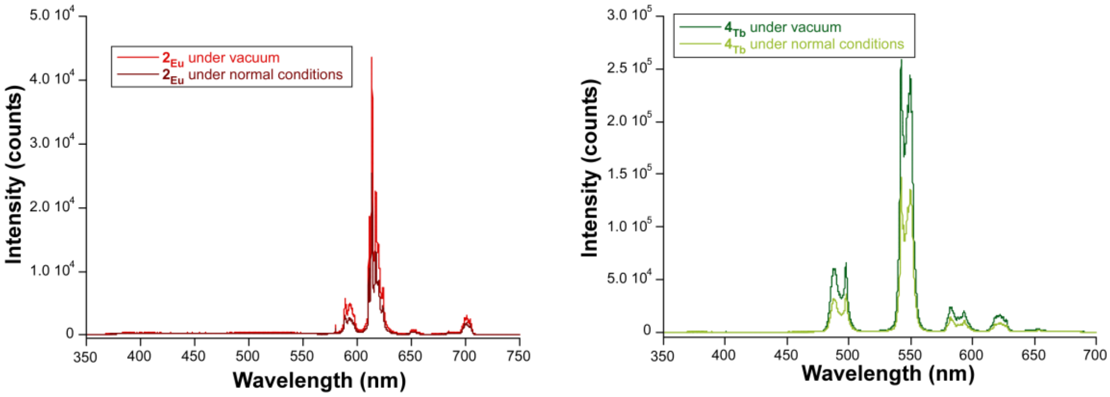

Figure S35. Emission spectra of the free $H_2$5Meip ligand sample taken at room temperature at λ_{ex} = 325 nm.

S7. Sensing experiments.

Given the good photoluminescence performance of compounds **2$_{Eu}$** and **4$_{Tb}$**, we have checked their stability and emission capacity suspended in water. These measurements were performed with the Varian Cary Eclipse spectrophotometer as specified in the Experimental section of the manuscript.

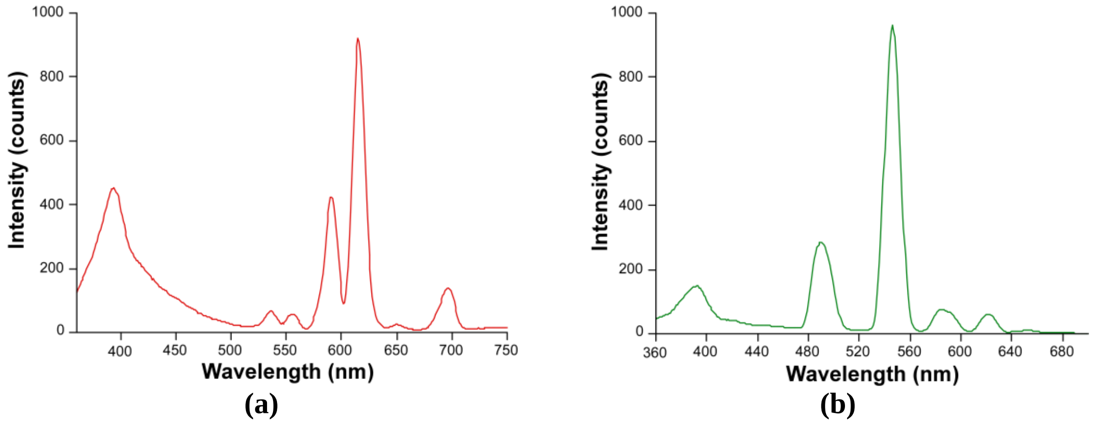

Figure S36. Emission spectra of compounds **(a) 2$_{Eu}$** and **(b) 4$_{Tb}$** suspended in water at room temperature.

Moreover, to confirm the stability in water of these two compounds, powders were filtered from the solution and once dried, a PXRD pattern was measured.

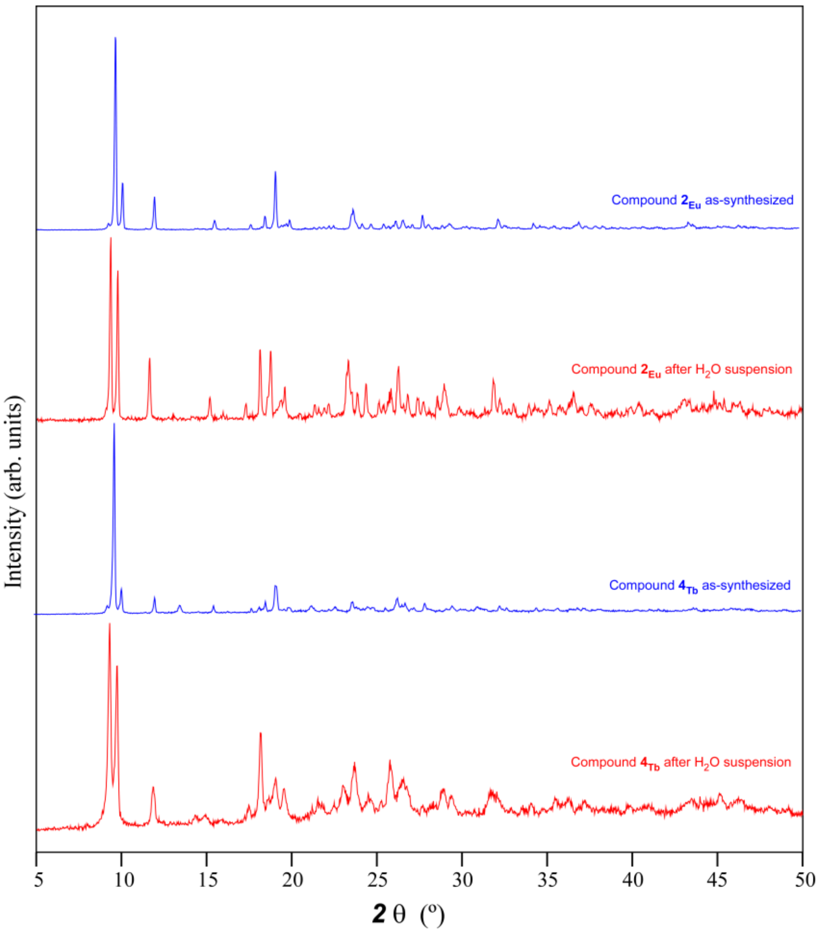

Figure S37. Comparison of the PXRD patterns for the as-synthesized and water suspended samples of compounds **2$_{Eu}$** and **4$_{Tb}$**.

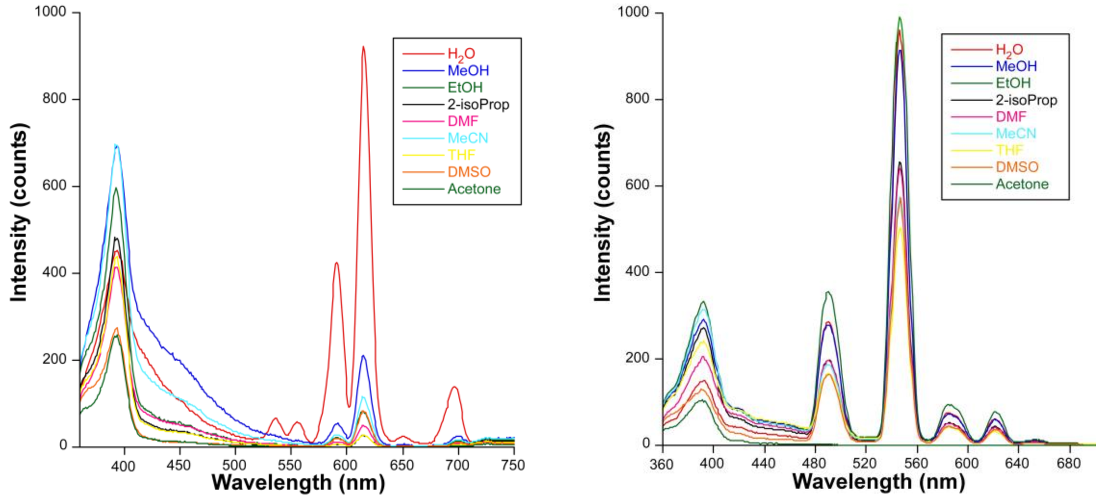

Figure S38. Emission spectra of compounds **(a)** $\mathbf{2_{Eu}}$ and **(b)** $\mathbf{4_{Tb}}$ suspended in different solvents at room temperature.

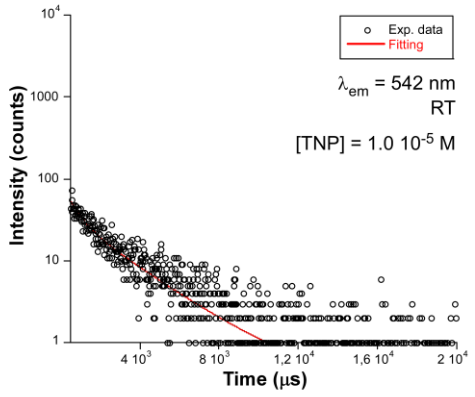

Figure S39. Emission decay curve of compound $\mathbf{4_{Tb}}$ suspended in water at a given concentration of TNP at room temperature.

After the titration of compound $\mathbf{4_{Tb}}$ with TNP in water, the stability of the sample was also ensured by its similar PXRD pattern.

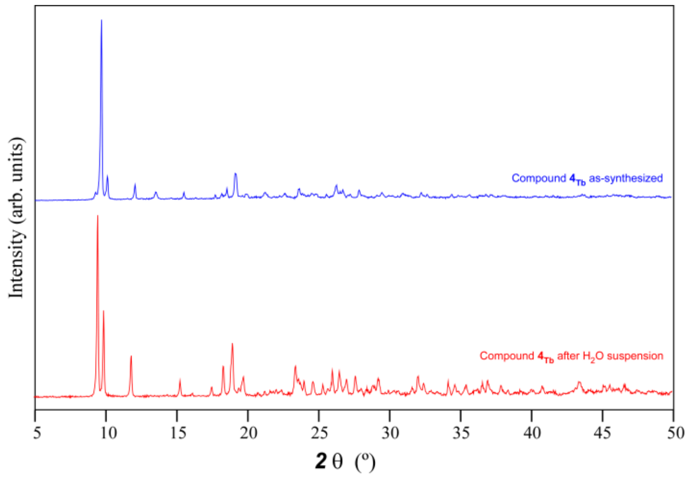

Figure S40. Comparison of the PXRD data for compound $\mathbf{4_{Tb}}$ for the as-synthesized sample and after water suspension titration with TNP.

A batch of suspensions containing compound **4$_{Tb}$** were prepared as detailed in the experimental section of the manuscript in which the concentration of TNP was gradually increased A Stern-Volmer analysis was conducted for the low- and high-concentration regimes to distinguish between the linear and curved regimes.

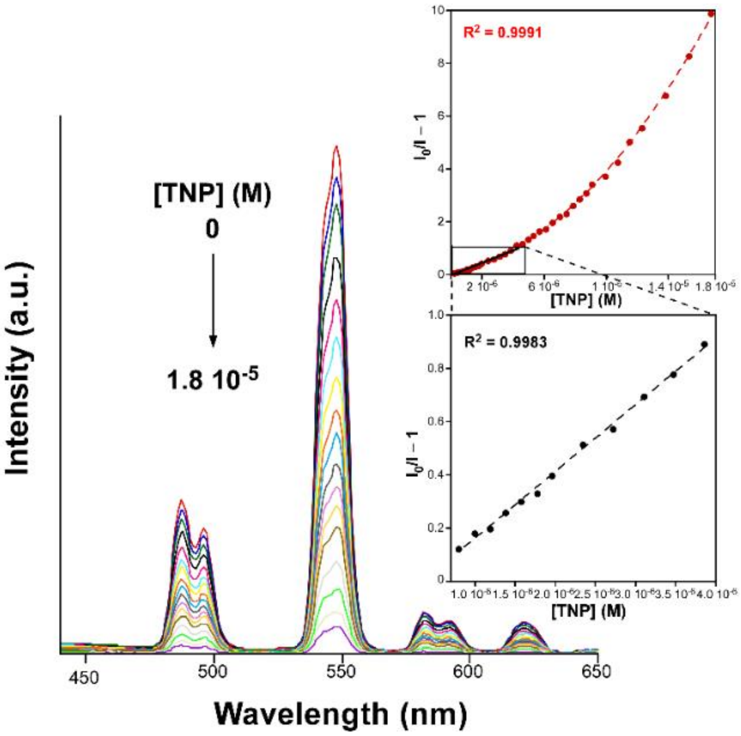

Figure S41. Room temperature emission spectra of **4$_{Tb}$**@H_2O in which variable concentration of TNP is added (λ_{ex} = 310 nm). Insets show the Stern-Volmer plots for the titration experiment with the whole studied concentration region (top) and the low concentration linear regime (bottom).

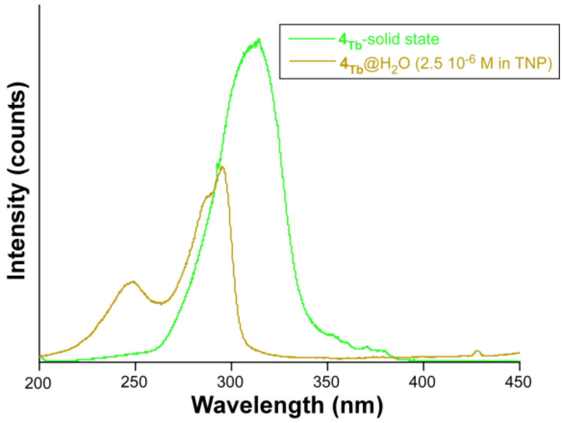

Figure S42. Comparative excitation spectrum of compound **4$_{Tb}$** in solid state and in water suspension containing a low concentration of TNP.

In order to improve the selectivity between 3-NP and TNP molecules for the luminescence quenching experiment, a comparative was also accomplished using MeOH as solvent. Below are the emission spectra recorded in solutions containing both target molecules dissolved in small concentration.

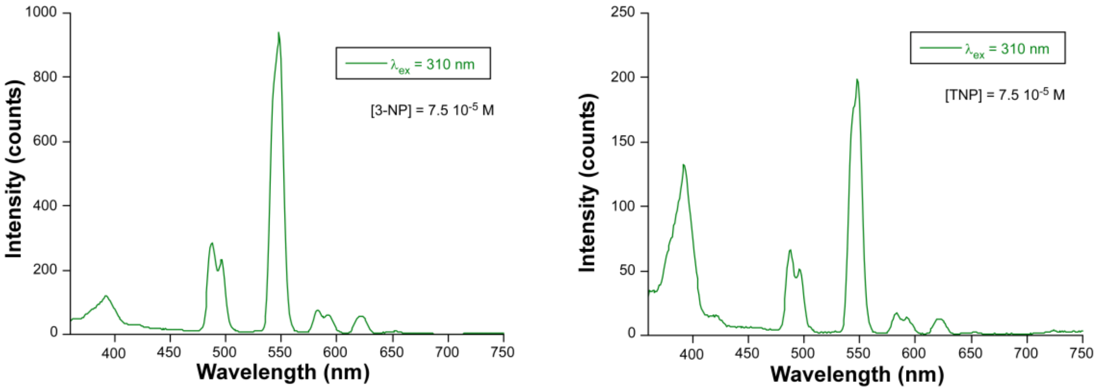

(a) **(b)**

Figure S43. Emission spectra for **4_{Tb}**@MeOH suspensions containing the corresponding nitroaromatic molecule dissolved: **(a)** 3-NP and **(b)** TNP.

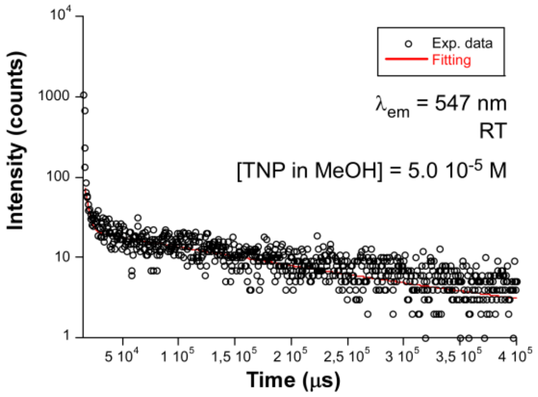

Figure S44. Emission decay curve of the suspension **4_{Tb}**@MeOH at a given concentration of TNP at room temperature.

On another level, PL sensing properties of compound **4_{Tb}** in aqueous suspensions have been analyzed for detecting TNP given its large quenching compared to other studied nitroaromatic molecules. The linear dependence of the intensity according to the concentration for the low concentration regime allows data be fitted to the Stern-Volmer equation:

$$I_0/I = 1 + k_{SV}[Q] \qquad \text{(eq. 1)}$$

On the contrary, the evaluation of the whole concentration regime shows a non-linear curve indicating the simultaneous occurrence of dynamic and static quenching. Accordingly, the curve has been fitted to a 2nd order polynomial expression which accounts for both processes:[1]

$$I_0/I = (1 + k_{SV}[Q])(1 + k_a[Q]) = 1 + (k_{SV} + k_a)[Q]) + k_{SV}k_a[Q]^2$$

where k_{SV} and k_a stand for the constants involving dynamic and static quenching processes. Best fitting results for the polynomial expression give:

$$C = 0.0176 \quad (k_{SV} + k_a) = 145839 \quad k_{SV}k_a = 2.0 \times 10^{10}$$

In the case of the sensing experiment conducted in MeOH, the best fitting results were:

$$C = 1.0297 \quad (k_{SV} + k_a) = 44097 \quad k_{SV}k_a = 3.0 \times 10^{10}$$

[1] (a) J. V. Goodpaster and V. L. McGuffin, *Appl. Spectrosc.*, 1999, **53**, 1000–1008. (b) J. Kusba, V. Bogdanov, ,I. Gryczynski and J. R. Lakowicz, *Biophys J.*, 1994, **5**, 2024–2040.
